# Genetic and molecular basis of abnormal BOLD signaling variability in patients with major depressive disorder after electroconvulsive therapy

**DOI:** 10.1038/s41398-025-03330-6

**Published:** 2025-04-02

**Authors:** Siyu Fan, Yulin Zhang, Rui Qian, Jie Hu, Hao Zheng, Wentao Dai, Yang Ji, Yue Wu, Xiaohui Xie, Si Xu, Gong-Jun Ji, Yanghua Tian, Kai Wang

**Affiliations:** 1 Department of Neurology, the Second Affiliated Hospital of Anhui Medical University, Hefei, China; 2 Department of Neurology, the First Affiliated Hospital of Anhui Medical University, Hefei, China; 3 Department of Psychology and Sleep Medicine, the Second Affiliated Hospital of Anhui Medical University, Hefei, China; 4 The College of Mental Health and Psychological Sciences, Anhui Medical University, Hefei, China; 5 Collaborative Innovation Center of Neuropsychiatric Disorders and Mental Health, Hefei, China; 6 Institute of Artificial Intelligence, Hefei Comprehensive National Science Center, Hefei, China

**Keywords:** Depression, Clinical genetics

## Abstract

Electroconvulsive therapy (ECT) is an effective and rapid neuromodulatory intervention for treatment-resistant major depressive disorders (MDD). However, the precise mechanisms underlying their efficacies remain unclear. Resting-state functional magnetic resonance imaging (fMRI) data were collected from 84 individuals with MDD and healthy controls before and after ECT, and coefficient of variation of the BOLD signal (CVBOLD) analysis was combined with region of interest (ROI) functional connectivity (FC) analysis. To assess the reliability of the antidepressant mechanism of ECT, we analyzed the changes in CVBOLD in a separate cohort consisting of 35 patients with MDD who underwent ECT. Moreover, transcriptomic and neurotransmitter receptor data were used to reveal the genetic and molecular bases of the changes in CVBOLD. Patients with MDD who underwent ECT demonstrated increased CVBOLD in the left angular cortex and left precuneus. Following ECT, an increase in FC between the left precuneus and right lingual lobes was associated with improvements in Hamilton Depression Rating Scale (HAMD) scores. validation analysis consistently demonstrated similar changes in CVBOLD in two independent cohorts of patients with MDD. Moreover, these changes in CVBOLD were closely associated with thyroid hormone synthesis, oxidative phosphorylation, endocytosis, and the insulin signaling pathway, and were significantly correlated with the receptor/transporter density of serotonin and dopamine. These findings suggest that ECT modulates abnormal functions in the left angular cortex and left precuneus, leading to widespread changes in functional connectivity and neuroplasticity, especially in the default mode network, and exerts an antidepressant effect.

## Introduction

Major depressive disorder (MDD) is a major global mental health challenge and a leading cause of mental health-related disabilities worldwide [1]. According to a World Health Organization (WHO) assessment report on the global disease burden, depression is expected to become the world’s largest disease burden by 2030 [2]. Treatment is urgently required to mitigate the heavy and growing burden of MDD. Existing antidepressant treatments are limited by slow action, lack of efficacy, and adverse outcomes [3]. However, electroconvulsive therapy (ECT) is considered one of the best methods for treating MDD [4], and the remission rate can reach 50–60% [5]. The specific mechanisms underlying ECT-induced responses in MDD remain unclear. Thus, exploring these neural mechanisms may provide better insights into MDD and improve clinical outcomes.

ECT may be able to improve MDD symptoms by inducing neuroplasticity and reorganizing the functional interactions of brain regions or large-scale brain networks [6, 7]. Traditional resting-state functional MRI (rs-fMRI) studies aim to investigate neuronal activation-induced changes in blood oxygenation-level-dependent (BOLD) signals [8]. However, the BOLD signal showed intrinsic variability over time and between individuals [9, 10]. Consequently, rs-fMRI studies exploring the differences in physiological fluctuations between various populations may provide important insights into the flexibility and adaptability of brain activities. Coefficient of variation of the BOLD signal (CVBOLD) has been increasingly used as a biomarker for specific neurological or psychiatric disorders in clinical investigations [11, 12]. In contrast, functional connectivity (FC) can assess the synchronous activity between different brain regions and reflect the connection strength and integration of neural networks. Studies using FC to analyze rs-fMRI have identified several areas of disrupted coordination in patients with MDD [13]. By combining the CVBOLD and FC, it is possible to simultaneously understand the stability and dynamic changes in brain connectivity in patients with MDD after ECT.

The clinical efficacy of ECT varies markedly among individuals and this variability is associated with polymorphisms in several MDD-associated genes [14]. The brain regions sensitive to ECT were enriched in neuroplasticity- and neuroimmunity-related genes [15]. The public availability of the Allen Human Brain Atlas (AHBA) has bridged the gap between transcriptomic expression profiles and neuroimaging phenotypes [16]. Furthermore, the etiology of MDD is strongly linked to dysfunctional activity within the serotonin, dopamine D2 receptor, and noradrenaline (monoaminergic) transmitter pathways [17]. The region-specific plasticity induced by ECT may be related to the differential modulation of neurotransmitter signaling [18]. A recent study developed a comprehensive three-dimensional atlas of the entire brain, mapping 19 receptors and transporters from nine distinct neurotransmitter systems. This atlas was created using positron emission tomography data collected from over 1200 healthy participants [19]. Transcriptomic and neurotransmitter datasets are excellent tools for exploring the molecular mechanisms underlying the antidepressant effects of ECT.

This study aimed to investigate the genetic and molecular mechanisms of ECT in patients with MDD using voxel-based morphometry, transcriptomics, and neurotransmitter receptor data. We hypothesized that ECT may modulate abnormal functions in specific brain regions and that the synaptic plasticity induced by ECT may be related to specific metabolic pathways and regional densities of neurotransmitter-associated proteins (e.g., transporters and receptors). Therefore, we examined the pattern of CVBOLD changes following ECT in two independent MDD cohorts and investigated regional associations with genes implicated in neuroplasticity, as well as with neurotransmitter transporters and receptors.

## Methods

### Participants and clinical assessments

All participants met the DSM-IV criteria for MDD diagnosis. According to statistical power analyses, for a liberal threshold of 0.05, 12 subjects were required to achieve 80% power at the single-voxel level and double the number of subjects was needed to maintain this level of power at more realistic thresholds, correcting for multiple comparisons [20]. The model estimated parameters from real fMRI data and then used the parameters in simulation experiments to generate power curves. Thus, 42 patients with MDD from the Anhui Mental Health Center (AMHC) were enrolled and scanned at the University of Science and Technology of China (USTC) between February 2017 and December 2019. Notably, some of the participants in this study had already participated in a previous study [6]. The Anhui Medical University Ethics Committee gave their approval for this research, and all participants gave written consent. Clinical and magnetic resonance imaging (MRI) evaluations were performed at two distinct points: TP1 and TP2. The HC group underwent a similar assessment schedule but did not receive ECT treatment. The detailed exclusion criteria and assessment procedures are available in the Supplementary Materials.

### ECT procedure

At AMHC, a revised bifrontal ECT technique was introduced in accordance with the 2019 guidelines of the Chinese Association of Physicians for Electrical Shock and Nerve Stimulation. The procedure was performed using a Somatics Thymatron System IV Integrated ECT System (Lake Bluff, IL, USA) with a maximum output of 1008 mC. Anesthesia was induced with propofol and muscle relaxation during each session was achieved using succinylcholine. The details of the ECT are available in the Supplementary Materials.

### MRI data acquisition and MRI preprocessing

Both structural and functional MRI scans using a 3-T scanner (Discovery GE750w; USTC). During scanning, the participants were asked to remain vigilant with their eyes closed. Functional images consisted of 217 echo-planar imaging volumes, with a repetition time (TR) of 2400 ms, an echo time (TE) of 30 ms, a 90° flip angle, a matrix size of 64 × 64, a field of view (FOV) of 192 × 192 mm^3^, and a slice thickness of 3 mm, totaling 46 slices (voxel size = 3 × 3 × 3 mm^3^). Additionally, anatomical images were captured with 188 slices, a TR of 8.16 ms, a TE of 3.18 ms, a 12° flip angle, an FOV of 256 × 256 mm^2^, a slice thickness of 1 mm, and a voxel size of 1 × 1 × 1 mm^3^. Functional data was preprocessed using the data processing assistant of the resting-state functional magnetic resonance imaging (rsfMRI) toolkit (DPARSF, http://rfmri.org/dpabi), a software package based on Statistical Parametric Mapping (SPM) version 12 (http://www.fil.ion.ucl.ac.uk/spm). Participants with a maximum displacement of less than 3 mm and angular motion of less than 3° were included in subsequent analyses. The detailed preprocessing steps are available in the Supplementary Materials.

### Algorithm of CVBOLD and FC analysis

For each participant, the CVBOLD map was calculated as the ratio of the standard deviation of the preprocessed BOLD time series to the mean of the preprocessed BOLD time series in each voxel. Next, we extracted the average CVBOLD in the gray matter. After the mixed-effects model analysis, the angular gyrus and precuneus were used as regions of interest (ROIs) for whole-brain FC analysis to explore the differences in whole-brain FC interactions in patients with MDD after ECT. Further details regarding the CVBOLD algorithm and FC analysis can be found in the Supplementary Materials.

### Validation analyses

To assess the reliability of the antidepressant mechanism of ECT, we analyzed the changes in CVBOLD in a separate cohort comprising 35 patients with MDD who underwent ECT. This cohort was recruited at the AMHC and scanned at Anhui Medical University (AHMU) between October 2012 and April 2017. All procedures except for the MRI scan parameters were performed as in the main cohort. Detailed MRI parameters are provided in the Supplementary Materials.

### Transcription-neuroimaging association analysis

The AHBA dataset (http://human.brain-map.org) bridges the gap between the regional changes in CVBOLD and transcriptomes [16]. Details regarding the AHBA dataset can be found in the Supplementary Materials. To evaluate the relationships between regional ΔCVBOLD t-values from 180 left hemisphere ROIs and the transcriptional activity of 10,027 genes, we employed the Partial Least Squares (PLS) correlation method. The first component (PLS1) represents the spatial map that explains the largest portion of the gene expression variance across ΔCVBOLD. In the discovery cohort, PLS1 accounted for 24% variance. The normalized PLS1 weights were ranked using a univariate one-sample Z tests.

### Gene enrichment analysis and protein–protein interaction analysis

Gene ontology (GO) and Kyoto encyclopedia of genes and genomes (KEGG) pathway analyses were performed using R software (version 4.3.1). The *clusterProfiler* package was used to identify significantly enriched GO terms and KEGG pathways. Statistical significance was set than 0.05. A PPI network was constructed using the STRING database (v11.0; https://cn.stringdb.org/) to explore the potential interactions among the proteins encoded by the 277 genes. Only interactions with a confidence score of 0.4 or higher were included. The network was visualized using Cytoscape (v3.10.0), where the nodes represent proteins and the edges denote the interactions between them. The node size was scaled according to the degree of connectivity, and the node color intensity was adjusted to reflect the magnitude of the node degree, with higher degrees represented by darker shading.

### Spatial correlation with neurotransmitter density

We used open-source PET data from unrelated control groups to construct distribution maps of the D2 receptor, inhibitory (5-HT1A and 5-HT1B) and excitatory (5-HT2A) 5-HT receptors, and serotonin transporter (5-HTT) [21, 22]. We then examined the linear correlations of space between PET and CVBOLD maps at individual locations, with significance corrected using the spin test (*pspin* < 0.05), a method based on angular permutations of spherical projections onto the cortical surface.

### Multivariate model

A multiple linear regression model was used to examine the effects of various neurotransmitters on neuroplasticity. The dependent variable, ΔCVBOLD, and the predictors (5HT1A, 5HT1B, 5HT2A, 5HTT, and D2 receptor) were first Z-transformed and then averaged separately across 180 ROIs in the left hemisphere, based on the HCP-MMP 1.0 atlas. Subsequently, we calculated the squared partial correlation between CVBOLD and each predictor. To determine the significance of the variance explained by the neurotransmitters, we compared these results with 10,000 spatially contiguous permuted null models [23].

## Results

### Demographics and clinical results

The demographic and clinical data of all the participants are summarized in Tables 1 and 2. In the USTC cohort, no significant distinctions between MDD patients and HC were observed in terms of age (t = −1.64, *p* = 0.104) or sex (χ2 = 0.34, *p* = 0.558). The severity of depressive symptoms significantly improved after ECT (paired t-test: t = 17.86, df = 41, *p* < 0.001), as evidenced by a decrease in HAMD scores among patients with USTC MDD. Additionally, the HAMD score also decreased significantly post-ECT (t = 16.14, *p* < 0.001).

**Table 1:** Demographic and clinical information of the MDD patients and Healthy controls in the USTC Cohort.

	MDD patients (Cohort USTC)	Healthy controls (Cohort USTC)	*p* value
Sample size	42	42	
Age (years)	39.29 ± 12.28	35 ± 11.61	0.104
Sex (male/female)	6/36	8/34	0.558
HAMD scores (ECT before)	25.57 ± 5.51	1.79 ± 1.99	<0.001
HAMD scores (ECT after)	7.26 ± 5.26	-	
MMSE scores (ECT before)	28.63 ± 1.79	29.51 ± 1.08	<0.05
MMSE scores (ECT after)	28.18 ± 2.05	-

**Table 2:** Comparison of the demographic and clinical information of the MDD patients in the USTC and AHMU Cohort.

	MDD patients (Cohort USTC)		MDD patients (Cohort AHMU)		*p* value
Age (years)	39.29 ± 12.28		39.94 ± 11.8		0.813
Sex (male/female)	6/36		12/23		0.036
HAMD scores	ECT before 25.57 ± 5.51	ECT after 7.26 ± 5.26	ECT before 23.29 ± 4.27	ECT after 4.74 ± 4.41	0.122
MMSE scores	ECT before 28.63 ± 1.79	ECT after 28.18 ± 2.05	ECT before 24.25 ± 10.25	ECT after -	0.355

*USTC* University of Science and Technology of China, *AHMU* Anhui Medical University, *MDD* major depressive disorder, *ECT* electroconvulsive therapy, *TP* time point, *HAMD* hamilton depression rating scale, *MMSE* mini-mental state examination.

### CVBOLD analysis

**Fig. 1:**
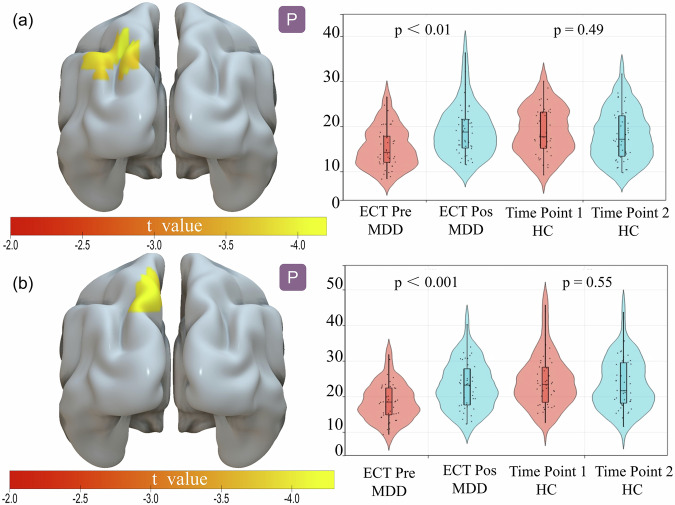
The change of CVBOLD after ECT in MDD in the main cohort of USTC. **a** The mean CVBOLD values of the left angular in MDD and HC groups before and after ECT; **b** The mean CVBOLD values of the left precuneus in MDD and HC groups before and after ECT. Pre pre-treatment, Pos post-treatment.

In the USTC cohort, there was a significant interaction effect between the group and TP in the left angular cortex and left precuneus (*p* < 0.05, with multiple comparisons adjusted using family wise error (FWE)). Post-hoc analysis revealed that the CVBOLD of the left angular gyrus in patients with MDD at TP2 was significantly enhanced compared to that at TP1 (t = −6.85; *p* < 0.001). Subsequently, the MDD and HC groups were compared at TP1 and HC demonstrated a higher CVBOLD in the left angular gyrus (t = −3.31; *p* < 0.001). Moreover, when comparing patients with MDD at TP1 and TP2 in the left precuneus gyrus, ECT was observed to improve the CVBOLD in the latter group (t = −6.44; *p* < 0.001). At TP1, the CVBOLD of the left precuneus in HC was higher than that in patients with MDD (t = −4.33; *p* < 0.001). No noteworthy divergence was noticed in CVBOLD in the left angular gyrus or left precuneus in either HC or patients with MDD at TP2. The CVBOLD caused by ECT in the USTC group is shown in Fig. 1 and Tables S1 and S2.

### Changes in FC strength

**Fig. 2:**
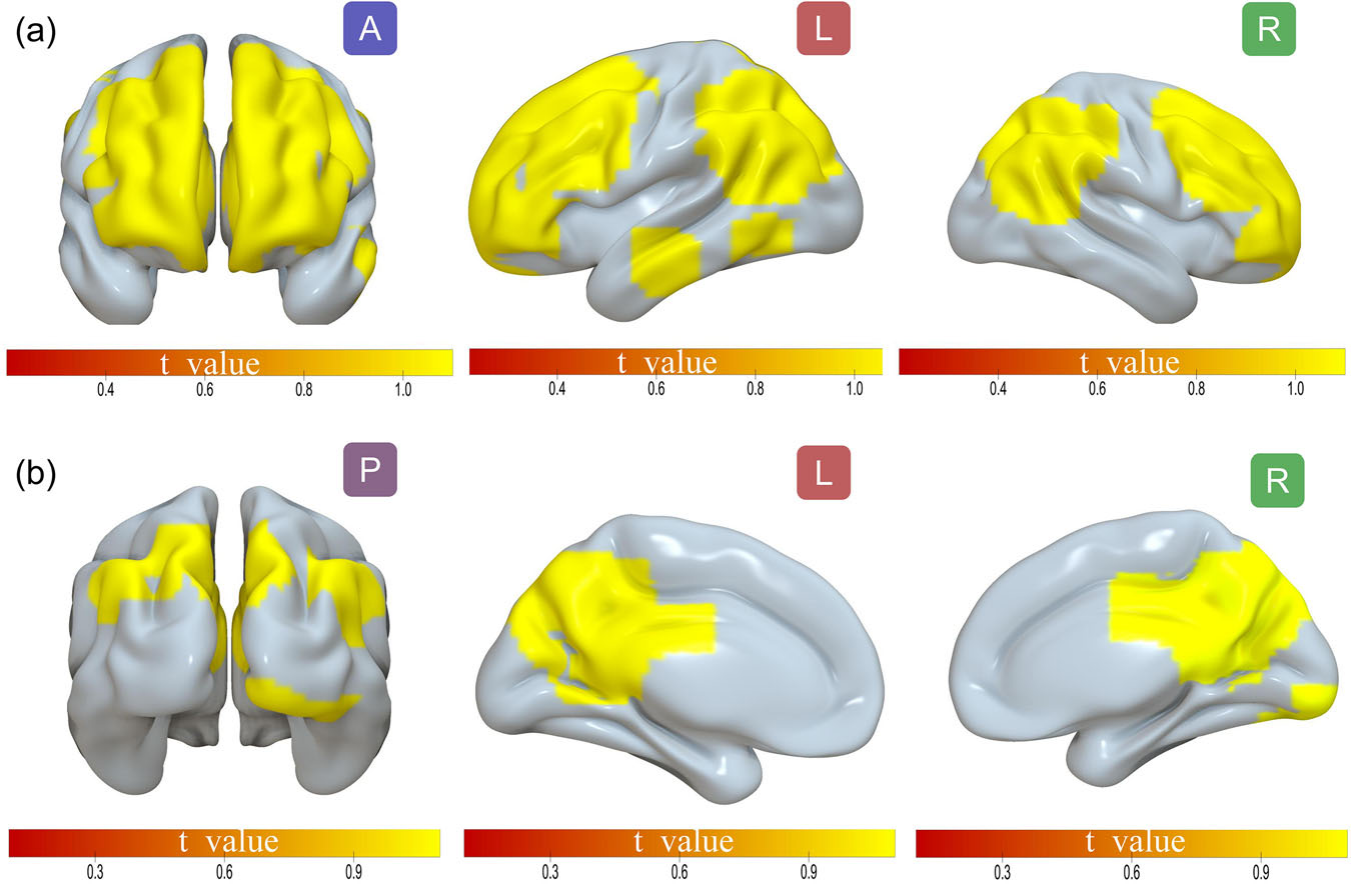
The change of FC strength after ECT in MDD in the main cohort of USTC. **a** A significant interaction effect for left angular between the group and TPs in the specific brain area; **b** A significant interaction effect for left precuneus between the group and TPs in the specific brain area.

ECT of the USTC sample augmented the functional link between the left angular and left precuneus. Guarantee the dependability of the mass and eliminate the impact of magnetic resonance artifacts. The left angular results revealed eight clusters with a significant ECT treatment effect (*p* < 0.05, with multiple comparisons adjusted for using Family Wise Error (FWE)). In addition to the left precuneus, seven clusters were found in the left middle temporal gyrus, left inferior temporal gyrus, right medial orbital frontal gyrus, right middle frontal gyrus, left middle frontal gyrus, right superior frontal gyrus, and bilateral precuneus. These clusters were located in the right lingual, occipital, bilateral precuneus, and bilateral angular and left superior parietal lobes. The FC strength induced by ECT in the USTC cohort is shown in Fig. 2, Table S3 and S4.

### Correlations analyses

**Fig. 3:**
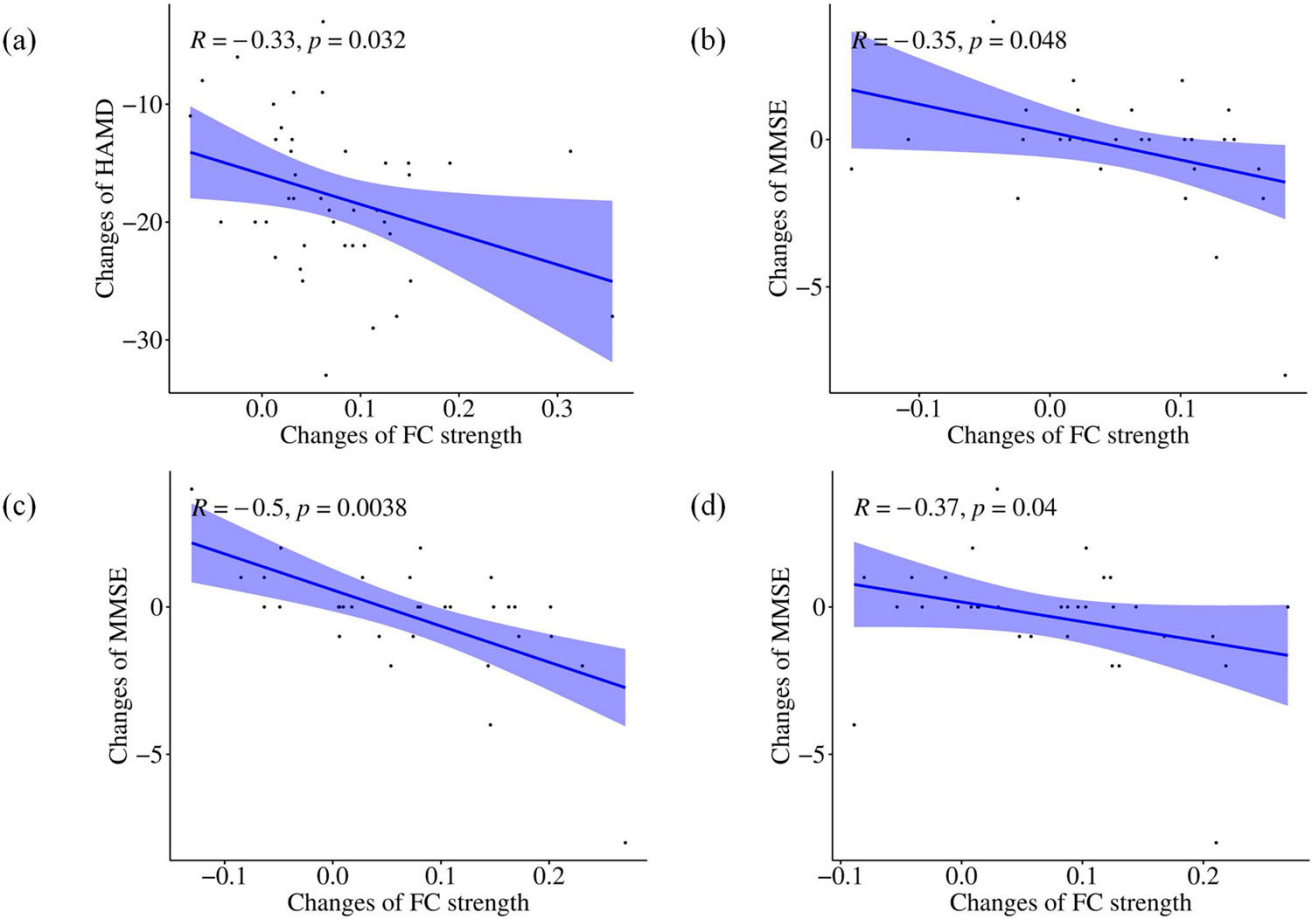
The correlations analyses between a change of FC strength and a change of HAMD and MMSE in MDD in the main cohort of USTC. **a** The increased FC strength of the left precuneus with the right lingual gyrus was negatively correlated to the changes of HAMD in MDD; **b** The increased FC strength of the left precuneus with the right precuneus was negatively correlated to the changes of MMSE in MDD; **c** The increased FC strength of the left precuneus with the left superior Parietal lobes was negatively correlated to the changes of MMSE in MDD; **d** The increased FC strength of the left angular with the left precuneus was negatively correlated to the changes of MMSE in MDD. lp left precuneus, rp right precuneus, lsp left superior parietal lobes, la left angular.

We found no correlation between CVBOLD values and changes in HAMD or MMSE values. However, we found that heightened FC between the left precuneus and right lingual gyrus was linked to HAMD scores (*p* = 0.032, *R* = −0.33, *p*_FDR_ = 0.128) after ECT. Additionally, a negative association was observed between FC values in the left precuneus and changes in MMSE scores in the left precuneus (*p* = 0.048, *R* = −0.35, *p*_FDR_ = 0.192) and left superior parietal lobes (*p* = 0.0038, *R* = −0.5, *p*_FDR_ = 0.015). Moreover, elevated FC values in the left angular precuneus and right precuneus were inversely related to MMSE changes (*p* = 0.040, *R* = −0.37, *p*_FDR_ = 0.160). The results of the correlation analyses are shown in Fig. 3.

### Validation analyses

The independent cohort of 35 patients with MDD who underwent ECT at the AHMU showed a pattern similar to CVBOLD abnormalities to that of the main USTC cohort. At the AHMU, the HAMD scores of patients with MDD significantly declined after ECT (t = 16.14, *p* < 0.001). Patients also showed lower CVBOLD in the left and right angular gyri. The CVBOLD induced by ECT in the AHMU cohort is shown in Fig. S1, Table S5 and S6.

### Gene enrichment and PPI analyses

The PLS1 weighted gene expression map spatially correlated with the t-map (Pearson’s r(150) = 0.49, *p* < 0.0001; Fig. S2). 199 PLS1+ (Z > 5) and 78 PLS1− (Z < − 5) (all *p*_FDR_ < 0.005) positively (or negatively) weighted gene expressions were overexpressed (or under-expressed) as increased (or decreased) regional changes in CVBOLD, respectively. Overall, 277 genes showed regional changes in the CVBOLD gene list of individuals with MDD.

**Fig. 4:**
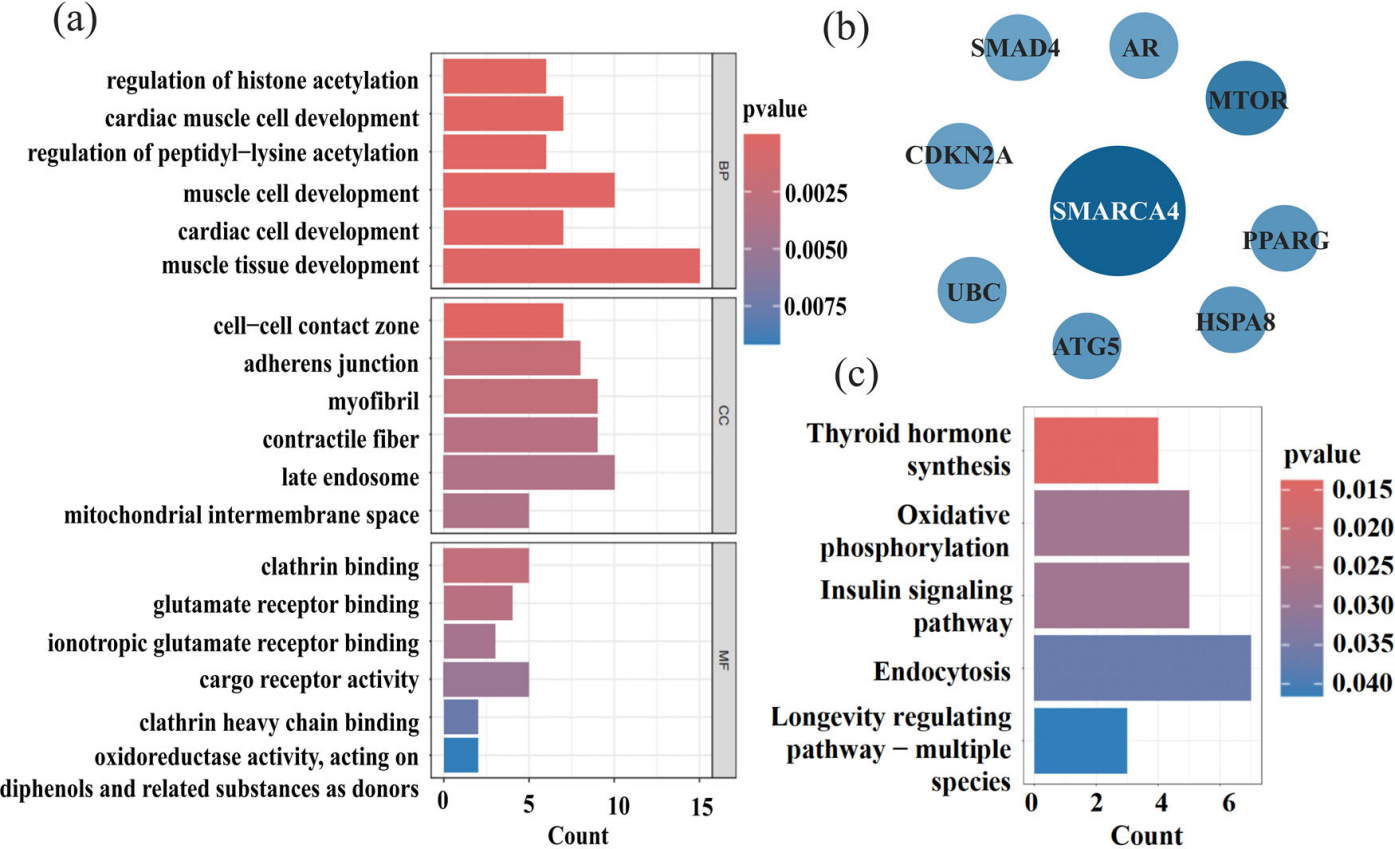
GO, KEGG and PPI network of the 277 genes. **a** Bar plot of the significant Gene Ontology (GO) categories enriched among the 277 genes. The y-axis represents specific GO terms across three main categories: Biological Processes (BP), Cellular Components (CC), and Molecular Functions (MF). The x-axis quantifies the enrichment significance, indicated by the count of genes associated with each term. **b** PPI analysis of the hub genes associated with changes of CVBOLD after ECT. **c** Bar plot of the significant KEGG pathways enriched among the 277 genes.

GO analysis revealed significant enrichment in processes such as the regulation of histone acetylation, cell-cell contact zone, clathrin binding, and glutamate receptor binding. KEGG pathway analysis highlighted key pathways including thyroid hormone synthesis, oxidative phosphorylation, the insulin signaling pathway, endocytosis, the longevity regulating pathway, and efferocytosis. The constructed PPI network comprised multiple clusters of highly interconnected proteins, suggesting the presence of functional modules within the network. Nodes with higher degrees, indicative of key hub proteins, were identified and visualized with increased size and darker colors in the network diagram. These hub proteins are potential candidates that may play key roles in the regulation of ECT efficacy (Fig. 4 and Fig. S3).

### Association with neurotransmitter density maps

**Fig. 5:**
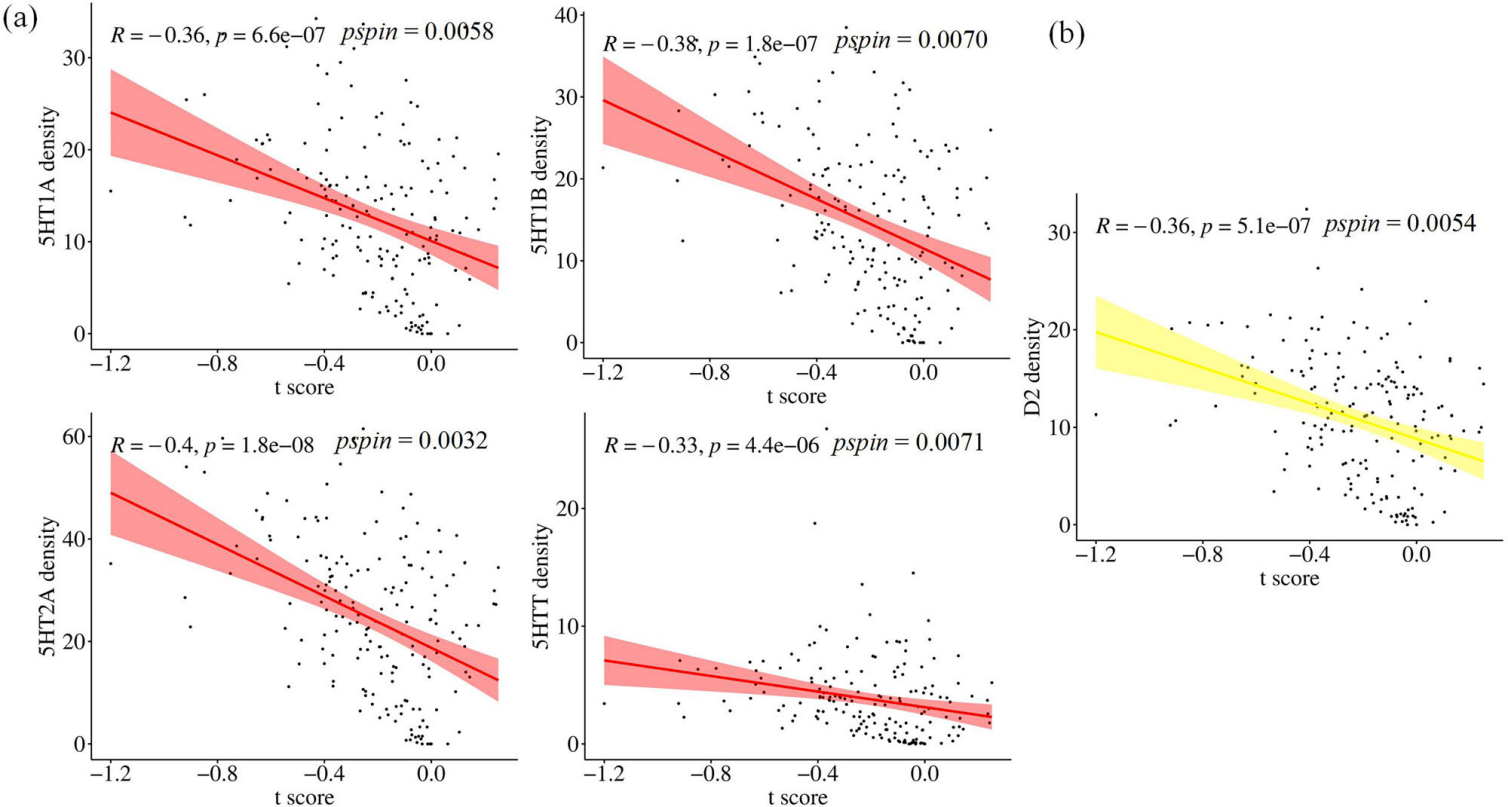
Association with nr density maps. **a** Neurotransmitter receptors and transporters density maps of serotonin were significantly negatively correlated with t-map of CVBOLD changes after ECT (*p* < 0.05, Bonferroni corrected). **b** Neurotransmitter receptors density maps of dopamine were significantly negatively correlated with t-map of CVBOLD changes after ECT (*p* < 0.05, Bonferroni corrected).

To identify the molecular basis of ECT, a spatial correlation between the changes in CVBOLD and neurotransmitter density maps was performed. The changes of CVBOLD in MDD patients after ECT compared to before treatment were negatively correlated with the receptor/transporter densities of serotonin (5-HT1A: *R* = −0.36, *pspin* = 0.0058; 5HT1B: *R* = −0.38, *pspin* = 0.0070; 5HT2A: *R* = −0.40, *pspin* = 0.0032; 5HTT: *R* = −0.33, *pspin* = 0.0071) and dopamine (D2: *R* = −0.36, *pspin* = 0.0054)(Fig. 5).

### Multivariate model combining neurotransmitter contributions

**Fig. 6:**
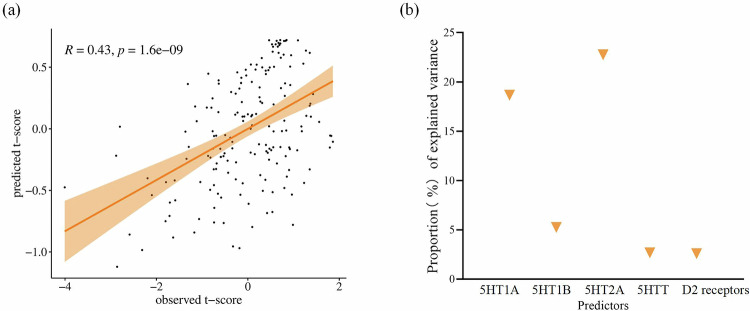
Multiple linear regression model relating neurotransmitter/receptor density with CVBOLD changes. **a** Fitted map of CVBOLD changes and scatter plot of predicted versus observed values. **b** Proportion (%) of variance explained by each individual predictor as calculated using the partial correlation coefficient with CVBOLD changes.

To examine the contributions of neurotransmitters to the observed CVBOLD changes, we constructed a multiple linear regression model with the predicted measures, including 5HT1A, 5HT1B, 5HT2A, 5HTT, and D2 receptor maps. The model explained 21% of the variance in ΔCVBOLD (*R*² = 0.21, F = 9.13, *p* = 9.9204e-08). All individual factors significantly predicted the variance in the CVBOLD changes (Table S7 and Fig. 6).

## Discussion

This study combined CVBOLD, FC, transcriptomics, and neurotransmitter receptor density maps to reveal the genetic and molecular mechanisms underlying ECT. The neuronal activity of local brain regions was mirrored in the CVBOLD. Our results indicate that ECT can improve the CVBOLD of the left angle and left precuneus, indicating that ECT can improve the stability of the BOLD signal in local brain regions and make it more active to some extent between these specific cerebral regions. In this study, ECT improved symptoms in patients with depression by increasing the regional brain activity of the left angular gyrus and left precuneus. At the junction of the temporal, occipital, and parietal lobes lies the angular, which is deemed a critical conduit for conveying and unifying data. The angle and precuneus appear as cross-modal regions among many brain regions, integrating multiple sensory information to understand events, manipulate mental representations, and redirect attention to relevant information [24].

The angular gyrus, precuneus, and portions of the frontal lobe are the key regions in the default mode network (DMN), a widespread and distributed system. The DMN typically shows decreased activity during tasks but becomes more active when the body is at rest. It plays a role in several cognitive functions, including semantic understanding, reading comprehension, numerical reasoning, and attention shifting [24]. They are involved in autobiographical memory processing, self-management, and social cognitive functions [25]. Various mental disorders, such as Alzheimer’s disease, schizophrenia, and depression [26], are associated with DMN abnormalities. Several studies have shown that structural and functional alterations in the angular gyrus are associated with depression. In a functional network analysis comparing 52 patients with first-episode MDD and 40 healthy controls, individuals with depression exhibited reduced functional connectivity strength (FCS) in the left angular gyrus [27]. Moreover, the precuneus is a key region of the DMN implicated in self-processing and emotional regulation [28]. The precuneus of patients with MDD displays a structural decline in gray matter volume, and the intensity of depression is associated with the inferior frontal gyrus, precuneus gyrus, and prefrontal network [29]. Numerous investigations have demonstrated that both the angular gyrus and the precuneus are associated with the manifestation of various depressive symptoms. Consequently, they are often used as targets and indicators in depression treatment research, contributing to antidepressant outcomes. Moreover, regions within the frontal and parietal lobes exhibit abnormal neuronal activity. Consistent with earlier findings, our study also identified a functional disruption between the DMN and frontoparietal network (FPN) in patients [30–32]. FPN participants initiated and adjusted cognitive control [33–35]. The functional activities of the FPN and its connection with the DMN are dysfunctional in terms of processing, managing, and controlling emotional stimuli [36–38]. Therefore, we observed augmented functional links between the DMN and FPN in patients with MDD who responded to ECT, implying that ECT may reorganize the functional activities or connections of the DMN and FPN, allowing patients to break away from contemplation and boost their emotional processing capacity [39–41].

In gene set PLS1, the enriched KEGG pathways included “thyroid hormone synthesis,” “oxidative phosphorylation,” “endocytosis” and “insulin signaling pathway.” Inflammation and altered energy metabolism are two pathways implicated in the pathophysiology of MDD [42]. Reduced synaptic signaling and functional connectivity are key features of MDD pathophysiology [43]. Endocytosis can reduce the production of proinflammatory cytokines in neurons [44]. Furthermore, endocytosis can affect synaptic plasticity, which may be related to alterations in the brains of patients after ECT [45]. In contrast, thyroid hormone synthesis, oxidative phosphorylation, and insulin signaling pathways are involved in neuronal energy metabolism. Notably, the DMN is one of the most energy-intensive brain networks, which may make it more sensitive to a lack of organismal energy [46]. First, thyroid hormones remodel neural circuits in the cerebral cortex to coordinate energy metabolism, particularly those related to MDD [47]. Second, over 90% of the ATP in the brain is produced via the oxidative phosphorylation pathway [48]. Increased levels of inflammatory mediators in MDD can significantly interfere with mitochondrial oxidative phosphorylation and ATP production, ultimately leading to increased oxidative stress [49]. Mitochondria undergoing oxidative phosphorylation can modulate neurotransmitters in several ways, inducing the elimination of the postsynaptic dendritic spines involved in long-term inhibition, which further affects neuronal activity and synaptic plasticity [50]. Insulin is involved in the glucose metabolism. The insulin signaling pathway may be inhibited by an increase in reactive oxygen species (ROS), which cause insulin resistance and have been associated with various brain disorders, including depression [51]. Our findings also confirm that the antidepressant effects of ECT are related to the serotonin system and dopamine receptors, which is consistent with previous studies [15]. Deficiencies in monoamine neurotransmitters (e.g., such (5-HT) and dopamine (DA), are the underlying causes of MDD [52]. We also analyzed key genes in ECT for MDD and found that they were associated with signaling and synaptic remodeling. For example, mammalian target of rapamycin (mTOR) is a serine/threonine protein kinase that modulates cell proliferation, mortality, survival, and protein synthesis [53]. ECT may exert its antidepressant effects by participating in mTOR signaling. These genetic and molecular results should be interpreted cautiously; however, the transcriptomic and neurotransmitter data from patients with MDD were not included in the analysis. The findings of this study will be useful in understanding the genetic and molecular mechanisms by which ECT improves patients with MDD.

Despite these meaningful findings, several methodological issues need to be addressed in future studies. Similar to other studies using AHBA transcriptome data [54, 55], the data were available for a small sample size and sex imbalance, and four of the donors had only left hemispheric brain samples, which may introduce bias in our statistical analyses. Moreover, the transcriptomic and neurotransmitter data were derived from different participants, making it impossible to rule out individual variability. To validate our findings, future research should conduct transcription-neuroimaging association studies using both gene expression and neuroimaging data collected from the same individuals. Finally, only gray matter BOLD signals were reported in this study, whereas recent studies have shown that BOLD signals can be reliably detected in white matter and reflect neural activity [56–59]. Thus, future studies should supplement the analysis of white matter BOLD signals to further elucidate the effects of ECT on whole brain networks.

## Conclusion

A voxel-based analysis was conducted in this study, and it was observed that the left angular gyrus and left precuneus had heightened CVBOLD after ECT. Functional connectivity between the left angular precuneus and left precuneus was augmented using ECT. These findings highlight the important role of the DMN in regulating emotions and cognition in patients with MDD using ECT. Additionally, the therapeutic effects of ECT may result from the normalization of energy metabolism, neurotransmitter release, and synaptic plasticity to ameliorate depressive symptoms.

## Supplementary information

Supplementary Material

## Data Availability

All data generated or analysed during this study are included in this article. Further enquiries can be directed to the corresponding author.
