# Supplementary material for: Genetic and molecular basis of abnormal BOLD signaling variability in patients with major depressive disorder after electroconvulsive therapy
Source: Transl Psychiatry. 2025 Apr 2;15:117. doi: 10.1038/s41398-025-03330-6 (PMC11965524; doi:10.1038/s41398-025-03330-6)
Supplement: Supplementary file 1 — Supplementary Material [file 41398_2025_3330_MOESM1_ESM.docx]

Supplementary Material

**1.Methods**

**1.1 Participants and clinical assessments**

To guarantee uniformity, the following exclusion criteria were implemented: (1) those aged 18 or younger, (2) those who had undergone ECT in the past three months, (3) those with concurrent neuropsychiatric issues, and (4) those with any impediments to MRI scans or ECT administration. The HC group was subject to the same criteria.

TP1 was done within 24 hours before the MDD patient's initial ECT session, while TP2 was done 72 hours after the MDD patient's last ECT session. Before and after ECT treatment, clinical and MRI assessments were performed. The 17-item Hamilton Depression Rating Scale (HAMD) was used to assess depression severity, with remission defined as a HAMD score ≤ 7. Cognitive impairment was evaluated using the Mini-mental State Examination (MMSE), a global cognitive screening tool assessing orientation, attention, calculation, registration, recall, and language.

**1.2 ECT procedure**

A 0.9A constant current, a 1 ms brief pulse duration, and a frequency of 50 Hz were all employed in the modified ECT. Stimulation intensity for the first session was determined according to the age of each patient as standard (e.g., 50% for a 50-year-old patient). If no seizures were elicited, the percentage of energy was increased by 5% until an electroencephalographic seizure was observed. The first three ECT administrations occurred on consecutive days, and the remaining ECT administrations were conducted every other day with a break on weekends, for totally 6–12 sessions. MDD patients in both samples received antidepressants during ECT but the use of benzodiaz-epines, valproate, and/or lithium was terminated.

**1.3 MRI pre-processing**

The RS-fMRI data were pre-processed using the data processing assistant for the rs-fMRI toolkit, a software package based on the Statistical Parametric Mapping (SPM) software ((www.fil.ion.ucl.ac.uk/spm), and the rs-fMRI toolkit(http://www.restfmri.net)[1]. The Diffeomorphic Anatomical Registration through Exponentiated Lie Algebra (DARTEL) tool[2, 3] was employed to pre-process the process, which included discarding the initial five volumes to maintain magnetization equilibrium, slice-timing correction, realignment, co-registration to the structural images, and spatial normalization to the Montreal Neurological Institute (MNI) space. 24 Friston motion parameters were used as regressors, which included 6 head motion parameters, 6 head motion parameters onetime point before, and 12 corresponding squared items[4]. Additionally, the WM and CSF signals, determined by the prior standard masks in the SPM, were also regressed. Employing scrubbing of motion, time points with a framewise displacement (FD) greater than 0.5 were eliminated, as well as one-time points before and after the exclusion of motion-related prejudice confounding.

**1.4 AHBA dataset**

Brain-wide gene expressions were measured in six postmortem brains (age = 42.50 ± 13.38 years; male/female = 5/1) with 3702 spatially distinct samples (Supplemental Result 11.1 and Table S6). The AHBA dataset was processed according to Arnatkevic et al[5]. The six steps of preprocessing were as follows: (i) verifying probe-to-gene annotations using the Re-annotator toolkit[6]; (ii) filtering of probes (intensity-based filtering) that do not exceed background noise, excluding at least 50% of all samples across participants; (iii) probe selection, selecting the highest correlation to RNA-seq data; (iv) samples assignment to the D–K 308 atlas within 2 mm Euclidean distance of a parcel; (v) normalization of expression measures using a scaled robust sigmoid for each participant; and (vi) gene set filtering based on differential stability. Because the AHBA dataset included only two right hemisphere data, only the left hemisphere was considered in our analysis[5]. Thus, a mean of all samples in a region was calculated to obtain the matrix (152 regions × 10,027 gene expression levels) of transcriptional level values.

**1.5 Algorithm of CVBOLD and FC analysis**

The stability of the BOLD signal voxels was determined voxel-by-voxel across the entire brain. An isotropic Gaussian kernel (FWHM= 4 mm3) method was used to smooth each voxel, and the mean stability of the BOLD signal voxels in the brain within each brain mask or ROI was compared by mixed effects analysis. All statistical maps underwent cluster-level family-wise error (FWE) correction with a threshold of *p* < 0.05 (cluster-forming threshold at voxel-level *p* < 0.001). Then we investigated differences in large-scale functional interactions between MDD patients and healthy controls by analyzing whole-brain FC patterns for brain regions with significant differences in CVBOLD. These regions served as seed regions for the FC analysis, with Pearson correlation coefficients calculated between their mean time series and the time series for each voxel of the entire brain for each subject. Fisher’s z-transformation was applied to improve normality. Mixed effects analysis were conducted on the z values, controlling for head motion parameters, total gray matter volume, white matter volume, and cerebrospinal fluid volume as covariates. All statistical maps underwent cluster-level family-wise error (FWE) correction with a threshold of *p* < 0.05 (cluster-forming threshold at voxel-level *p* < 0.001).

**1.6 Validation analyses**

The parameters of the MRI were as follows: (1) rs-MRI: 240 volumes, TR = 2000 ms; 64×64 matrix; 220×220 mm² field of view; 33 continuous slices; one voxel = 3.4×3.4×4.6 mm³; (2) T1-weighted anatomical image: 188 slices, TR = 8.676 ms; TE = 3.184 ms; inversion time = 800 ms; flip angle = 8°; 256×256 mm² field of view; slice thickness = 1 mm; and voxel size = 1×1×1 mm³.

**References:**

1. Chao-Gan Y, Yu-Feng Z: **DPARSF: A MATLAB Toolbox for "Pipeline" Data Analysis of Resting-State fMRI**. *FRONT SYST NEUROSCI* 2010, **4**:13.

2. Chen X, Lu B, Yan CG: **Reproducibility of R-fMRI metrics on the impact of different strategies for multiple comparison correction and sample sizes**. *HUM BRAIN MAPP* 2018, **39**(1):300-318.

3. Ashburner J: **A fast diffeomorphic image registration algorithm**. *NEUROIMAGE* 2007, **38**(1):95-113.

4. Yan CG, Cheung B, Kelly C, Colcombe S, Craddock RC, Di Martino A, Li Q, Zuo XN, Castellanos FX, Milham MP: **A comprehensive assessment of regional variation in the impact of head micromovements on functional connectomics**. *NEUROIMAGE* 2013, **76**:183-201.

5. Arnatkeviciute A, Fulcher BD, Fornito A: **A practical guide to linking brain-wide gene expression and neuroimaging data**. *NEUROIMAGE* 2019, **189**:353-367.

6. Arloth J, Bader DM, Roh S, Altmann A: **Re-Annotator: Annotation Pipeline for Microarray Probe Sequences**. *PLOS ONE* 2015, **10**(10):e139516.

1. **Tables**

Table S1 The Bold signal stability of left angular and left precuneus

| Variables | PT (N=42) | | HC (N=42) | |
| --- | --- | --- | --- | --- |
|  | TP1 | TP2 | TP1 | TP2 |
| Left angular | 15.33±4.49 | 19.05±5.60 | 18.83±5.19 | 18.02±5.51 |
| Left precuneus | 18.46±5.11 | 23.02±1.01 | 24.42±7.31 | 23.49±1.08 |

Table S2 Differential brain regions induced by ECT

| Cluster number | Cluster size | Peak intensity | Peak MNI coordinates (x, y, z) | Brain regions (AAL) |
| --- | --- | --- | --- | --- |
| 1 | 52 | -4.897 | -33, -75, 51 | left angular |
| 2 | 39 | -4.696 | -6, -69, 60 | left precuneus |

Table S3 FC strength induced by ECT of left angular

| Cluster number | Cluster size | Peak intensity | Peak MNI coordinates (x, y, z) | Brain regions (AAL) |
| --- | --- | --- | --- | --- |
| 1 | 61 | -5.622 | -69, -15, -15 | left middle temporal gyrus |
| 2 | 47 | -4.527 | -57, -60, -15 | left inferior temporal gyrus |
| 3 | 56 | -3.589 | 3, 57, -12 | right medial orbital frontal gyrus |
| 4 | 1441 | -7.620 | 3, 42, 21 | right middle frontal gyrus  right superior frontal gyrus |
| 5 | 2194 | -6.779 | -3, 45, 21 | left middle frontal gyrus  left superior frontal gyrus |
| 6 | 16 | -3.406 | 12, 45, 3 | right superior frontal gyrus |
| 7 | 1505 | -5.560 | 36, -60, 51 | right precuneus  right angular |
| 8 | 1705 | -5.534 | -3, -36, 36 | left precuneus  left angular |

Table S4 FC strength induced by ECT of left precuneus

| Cluster number | Cluster size | Peak intensity | Peak MNI coordinates (x, y, z) | Brain regions (AAL) |
| --- | --- | --- | --- | --- |
| 1 | 29 | -3.999 | 42,-75,-21 | right lingual lobe |
| 2 | 25 | -4.145 | 15,-99,-15 | occipital Lobe |
| 3 | 310 | -5.553 | -3,-75,48 | left precuneus |
| 4 | 636 | -4.868 | 12,-60,21 | right precuneus |
| 5 | 16 | -4.561 | -48,-63,33 | left angular |
| 6 | 110 | -4.224 | 45,-60,36 | right angular |
| 7 | 36 | -4.081 | -30,-81,42 | left superior parietal lobe |

Table S5 The BOLD signal stability of left angular and right angular

| Variables | TP1 | TP2 | t value | p-value |
| --- | --- | --- | --- | --- |
| Left angular | 7.61±1.45 | 9.97±2.79 | -7.17 | <0.001 |
| Right angular | 7.22±1.52 | 9.30±2.48 | -6.02 | <0.001 |

Table S6 Differential brain regions induced by ECT

| Cluster number | Cluster size | Peak intensity | Peak MNI coordinates (x, y, z) | Brain regions (AAL) |
| --- | --- | --- | --- | --- |
| 1 | 63 | -4.585 | 48, -57, 36 | right angular |
| 2 | 30 | -3.877 | -39, -69, 39 | left angular |

Table S7 Results of multiple linear regression model predicting CVBOLD changes after ECT

| Predictor | β-value | Standard error | T value | % Explained variance | P value |
| --- | --- | --- | --- | --- | --- |
| Intercept | -1.10×10^-17^ | 0.067 | -1.63×10^-16^ | NA | 1 |
| 5HT1A | -0.35 | 0.070 | -4.98 | 12.24 | ＜0.0001 |
| 5HT1B | -0.40 | 0.069 | -5.70 | 15.44 | ＜0.0001 |
| 5HT2A | -0.42 | 0.068 | -6.20 | 17.75 | ＜0.0001 |
| 5HTT | -0.23 | 0.073 | -3.21 | 5.46 | 0.0022 |
| D2 receptors | -0.35 | 0.070 | -4.94 | 12.06 | ＜0.0001 |

Table S8 The CVBOLD-related gene list

| 1 | ABCF2 |
| --- | --- |
| 2 | ABHD12B |
| 3 | ACSF3 |
| 4 | ACTN2 |
| 5 | ADAMTSL1 |
| 6 | ADPRHL1 |
| 7 | ADRA1D |
| 8 | AFAP1L1 |
| 9 | AFAP1L2 |
| 10 | AGAP2 |
| 11 | AIFM1 |
| 12 | AKAP6 |
| 13 | AKAP8 |
| 14 | ALKBH3 |
| 15 | ALMS1P1 |
| 16 | AMDHD1 |
| 17 | ANKIB1 |
| 18 | ANKRD28 |
| 19 | ANKRD36B |
| 20 | ANKRD36BP1 |
| 21 | ANKRD36BP2 |
| 22 | ANKS1B |
| 23 | ANO7 |
| 24 | ANP32D |
| 25 | ANP32E |
| 26 | ARG2 |
| 27 | ARHGEF16 |
| 28 | ARID5A |
| 29 | ARL8B |
| 30 | ASGR1 |
| 31 | ASGR2 |
| 32 | ATG5 |
| 33 | ATG9A |
| 34 | ATP6V0A1 |
| 35 | AURKAIP1 |
| 36 | AVPI1 |
| 37 | BAIAP2L2 |
| 38 | BEX4 |
| 39 | BIRC2 |
| 40 | BNIP3 |
| 41 | C11orf87 |
| 42 | C1orf74 |
| 43 | C2CD4A |
| 44 | CA4 |
| 45 | CAMK1G |
| 46 | CASQ1 |
| 47 | CBLN2 |
| 48 | CCDC110 |
| 49 | CCDC85A |
| 50 | CCK |
| 51 | CDC37 |
| 52 | CDCA5 |
| 53 | CDH18 |
| 54 | CERS6 |
| 55 | CES1 |
| 56 | CIAPIN1 |
| 57 | CITED4 |
| 58 | CKB |
| 59 | CLPB |
| 60 | CNOT10 |
| 61 | COL5A2 |
| 62 | COMMD5 |
| 63 | CREB3L3 |
| 64 | CRLF1 |
| 65 | CRYBA2 |
| 66 | CRYBB1 |
| 67 | CTSL |
| 68 | DACH1 |
| 69 | DENND2D |
| 70 | DERL1 |
| 71 | DGCR5 |
| 72 | DMRTA2 |
| 73 | DNAJC8 |
| 74 | DNAJC9 |
| 75 | DNM3 |
| 76 | DTD1 |
| 77 | DUSP3 |
| 78 | DZANK1 |
| 79 | E2F1 |
| 80 | EGFL7 |
| 81 | EIF1AD |
| 82 | EIF2B2 |
| 83 | EPN2 |
| 84 | ERCC6L2 |
| 85 | FAIM2 |
| 86 | FAM173A |
| 87 | FBXL22 |
| 88 | FBXO40 |
| 89 | FGD1 |
| 90 | FHL2 |
| 91 | FLOT1 |
| 92 | FLOT2 |
| 93 | FNDC10 |
| 94 | FNDC3B |
| 95 | FOXQ1 |
| 96 | FREM3 |
| 97 | GCHFR |
| 98 | GEMIN7 |
| 99 | GFER |
| 100 | GFOD2 |
| 101 | GMPPA |
| 102 | GNG13 |
| 103 | GOLGA3 |
| 104 | GPATCH3 |
| 105 | GPR20 |
| 106 | GPX3 |
| 107 | GRASP |
| 108 | HARBI1 |
| 109 | HDAC9 |
| 110 | HEXB |
| 111 | HMGB3P1 |
| 112 | HMOX2 |
| 113 | HPS6 |
| 114 | HSPA12B |
| 115 | HSPA1L |
| 116 | HTR1E |
| 117 | HTR4 |
| 118 | IDH3B |
| 119 | IGFBPL1 |
| 120 | ISL1 |
| 121 | JAGN1 |
| 122 | KBTBD6 |
| 123 | KCNC2 |
| 124 | KCNJ11 |
| 125 | KCNS3 |
| 126 | KHDC1L |
| 127 | KIF17 |
| 128 | KMT2D |
| 129 | LCP2 |
| 130 | LDHB |
| 131 | LDLR |
| 132 | LGALS1 |
| 133 | LGR6 |
| 134 | LHPP |
| 135 | LIMK2 |
| 136 | LINC00167 |
| 137 | LINC01116 |
| 138 | LINC02018 |
| 139 | LMO4 |
| 140 | LOC100128239 |
| 141 | LOC100506476 |
| 142 | LOC105376360 |
| 143 | LRP1 |
| 144 | LRRC14 |
| 145 | LRRC32 |
| 146 | LRRC39 |
| 147 | LUC7L |
| 148 | LYRM4 |
| 149 | MAP3K2 |
| 150 | MAPK13 |
| 151 | MEDAG |
| 152 | MEG3 |
| 153 | METTL21A |
| 154 | MIEN1 |
| 155 | MRPL27 |
| 156 | MUC1 |
| 157 | MUM1L1 |
| 158 | MYADML2 |
| 159 | NAE1 |
| 160 | NAT10 |
| 161 | NAT6 |
| 162 | NBEAL2 |
| 163 | NBPF1 |
| 164 | NBPF3 |
| 165 | NDUFB2 |
| 166 | NECTIN1 |
| 167 | NETO2 |
| 168 | NLN |
| 169 | NPAT |
| 170 | NPRL2 |
| 171 | NRGN |
| 172 | NSG1 |
| 173 | NTMT1 |
| 174 | NUBP1 |
| 175 | OAS1 |
| 176 | ONECUT2 |
| 177 | PART1 |
| 178 | PCDHGA7 |
| 179 | PDE1B |
| 180 | PDYN |
| 181 | PEAK1 |
| 182 | PHKG2 |
| 183 | PICK1 |
| 184 | PLA2G4A |
| 185 | PMS2P3 |
| 186 | PMS2P5 |
| 187 | PMS2P7 |
| 188 | PNPLA1 |
| 189 | POMT1 |
| 190 | POP7 |
| 191 | PP12613 |
| 192 | PPIL4 |
| 193 | PPP1R14C |
| 194 | PRKAR1B |
| 195 | PRKCZ |
| 196 | PRR3 |
| 197 | PSIP1 |
| 198 | PSMA3-AS1 |
| 199 | PTPRK |
| 200 | RAB3B |
| 201 | RABEPK |
| 202 | RASGRF2 |
| 203 | RILP |
| 204 | RNF26 |
| 205 | RPA4 |
| 206 | RTN4IP1 |
| 207 | SCO2 |
| 208 | SDK1 |
| 209 | SERPINI2 |
| 210 | SET |
| 211 | SFPQ |
| 212 | SIAE |
| 213 | SLAMF7 |
| 214 | SLC17A4 |
| 215 | SLC22A18 |
| 216 | SLC35C1 |
| 217 | SLC35F1 |
| 218 | SLC35G3 |
| 219 | SLCO2A1 |
| 220 | SMAD4 |
| 221 | SMIM12 |
| 222 | SNORC |
| 223 | SPON2 |
| 224 | SPR |
| 225 | ST6GAL2 |
| 226 | ST8SIA3 |
| 227 | STARD4 |
| 228 | STEAP1 |
| 229 | STX19 |
| 230 | SURF4 |
| 231 | SV2B |
| 232 | SYNM |
| 233 | SYT13 |
| 234 | TDRD7 |
| 235 | TESPA1 |
| 236 | TET2 |
| 237 | TEX29 |
| 238 | TEX47 |
| 239 | THY1 |
| 240 | TIAM1 |
| 241 | TMEM134 |
| 242 | TMEM155 |
| 243 | TMEM183A |
| 244 | TMUB1 |
| 245 | TNNT2 |
| 246 | TOP1MT |
| 247 | TPBG |
| 248 | TPD52 |
| 249 | TPST2 |
| 250 | TRAM2 |
| 251 | TRAPPC1 |
| 252 | TRAPPC9 |
| 253 | TUBG1 |
| 254 | TYRP1 |
| 255 | UBALD1 |
| 256 | UBE2Q2P1 |
| 257 | UBE2R2 |
| 258 | UBE3D |
| 259 | UQCRC1 |
| 260 | UQCRH |
| 261 | VCL |
| 262 | VENTX |
| 263 | VPS18 |
| 264 | VPS25 |
| 265 | WDR24 |
| 266 | WDR43 |
| 267 | XRCC5 |
| 268 | YIPF2 |
| 269 | YIPF3 |
| 270 | YPEL5 |
| 271 | YWHAEP1 |
| 272 | ZNF302 |
| 273 | ZNF576 |
| 274 | ZNF584 |
| 275 | ZNF593 |
| 276 | ZNF638 |
| 277 | ZNF689 |

**3.Figures**


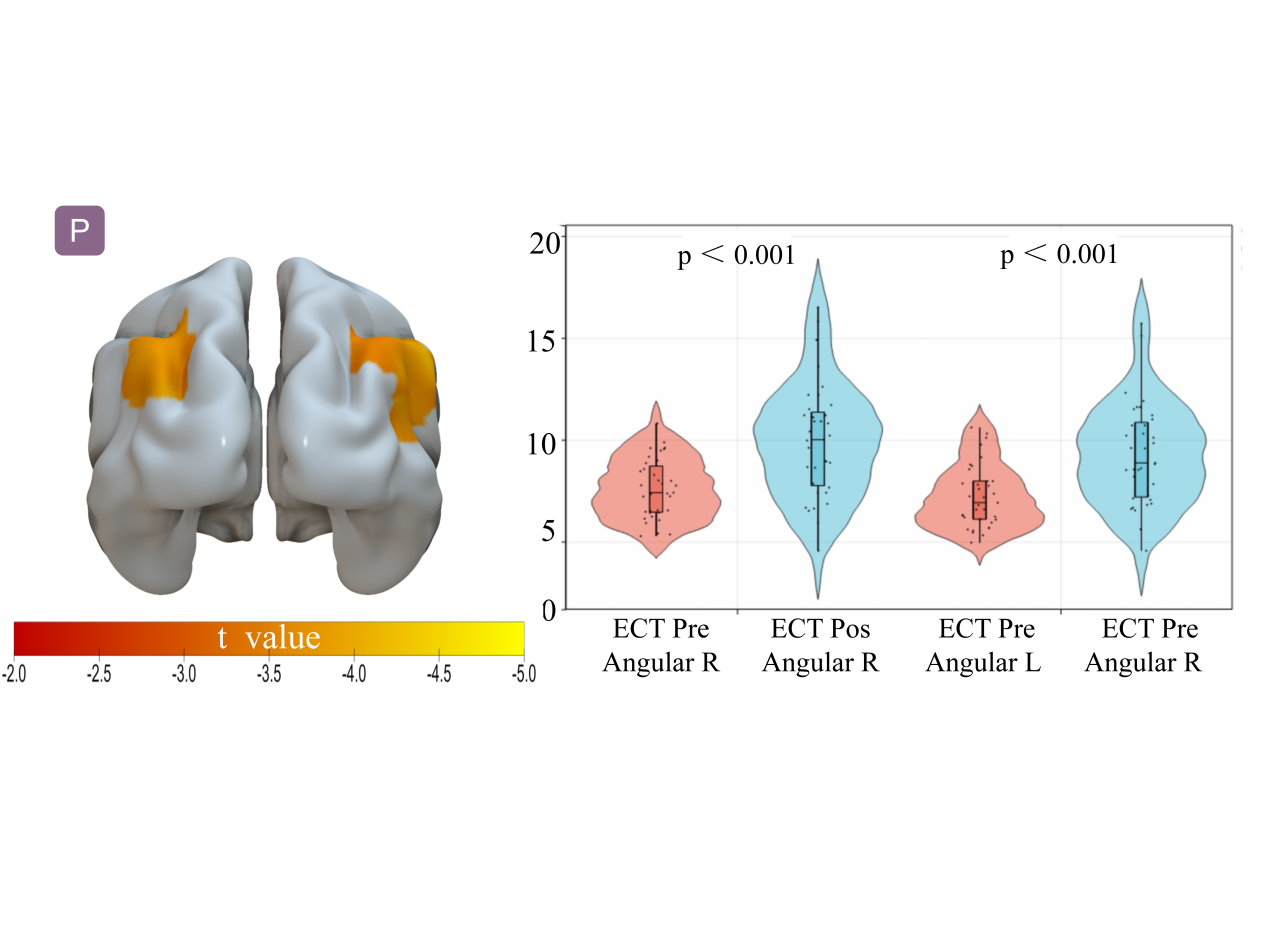


Fig.S1 The change of CVBOLD after ECT in MDD in the AHMU. (a) A significant interaction effect between the group and TPs in the bilateral angular cortex; (b) The mean CVBOLD values of the left AG in MDD at the two TPs; (c) The mean CVBOLD values of the right angular in MDD at the two TPs.


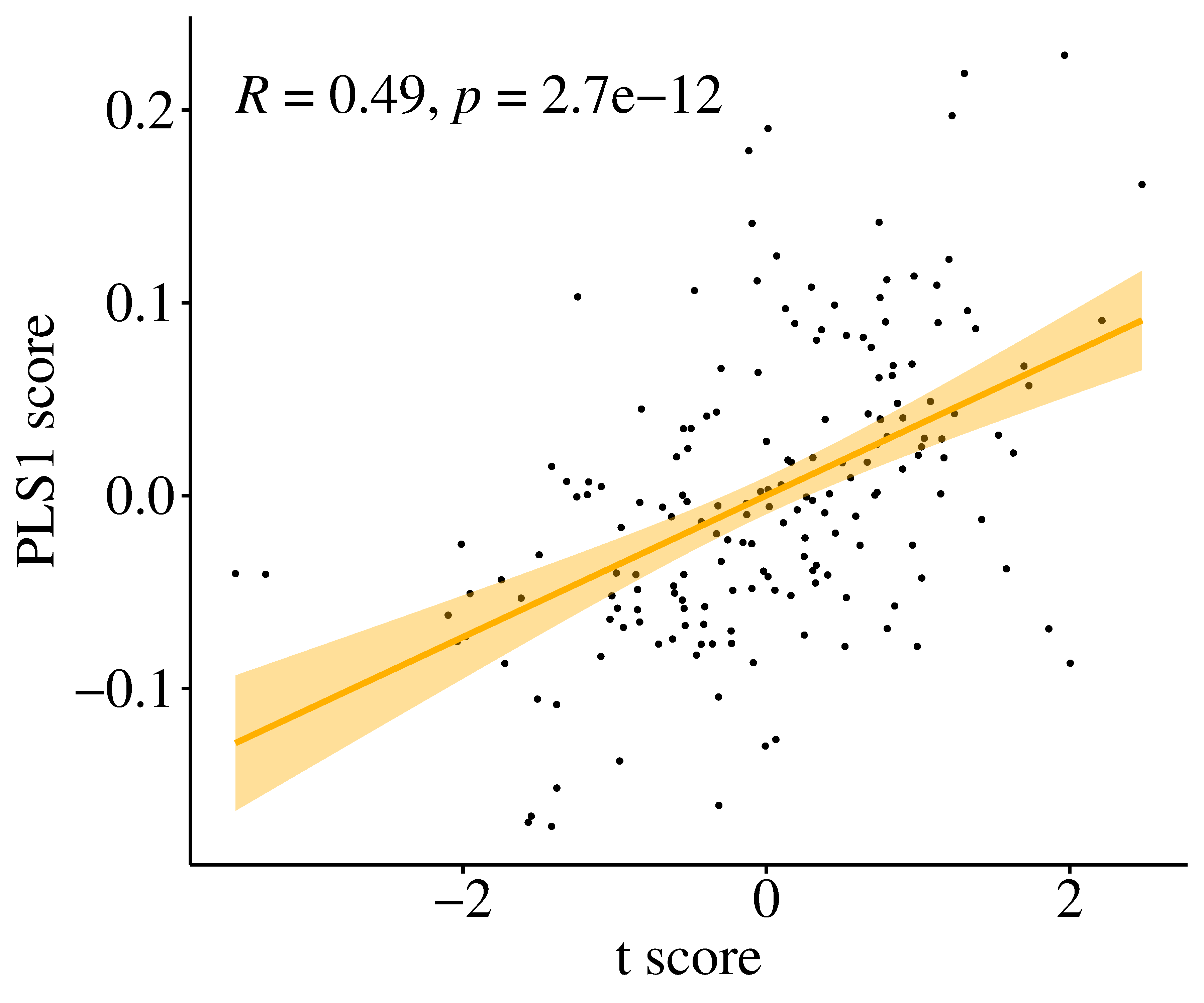


Fig.S2 Scatterplots of regional PLS1 scores and regional differences in t score were shown.


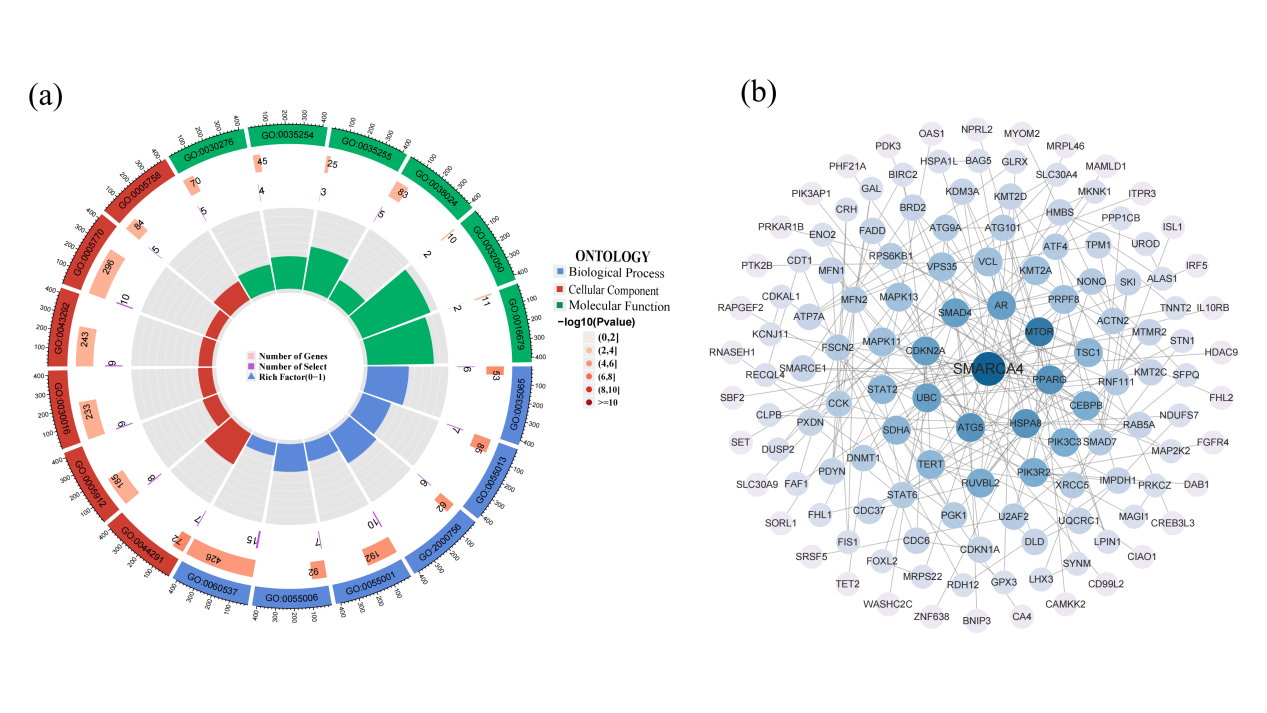


Fig.S3 GO and PPI network of the 277 genes. (a) Each segment of the circle plot represents a specific GO term. The color gradien denotes the -log10(p-value), with darker hues indicating higher significance levels of enrichment. The inner circle tracks show the 'Rich Factor', a ratio indicating the proportion of genes in the dataset that map to each GO term, with a scale from 0 to 1. (b) PPI analysis of the genes associated with changes of CVBOLD after ECT. Nodes represent proteins, scaled and colored according to their degree. Edges represent protein-protein interactions. Node size and color intensity increase with the degree, highlighting potential hub proteins with central roles in the network.


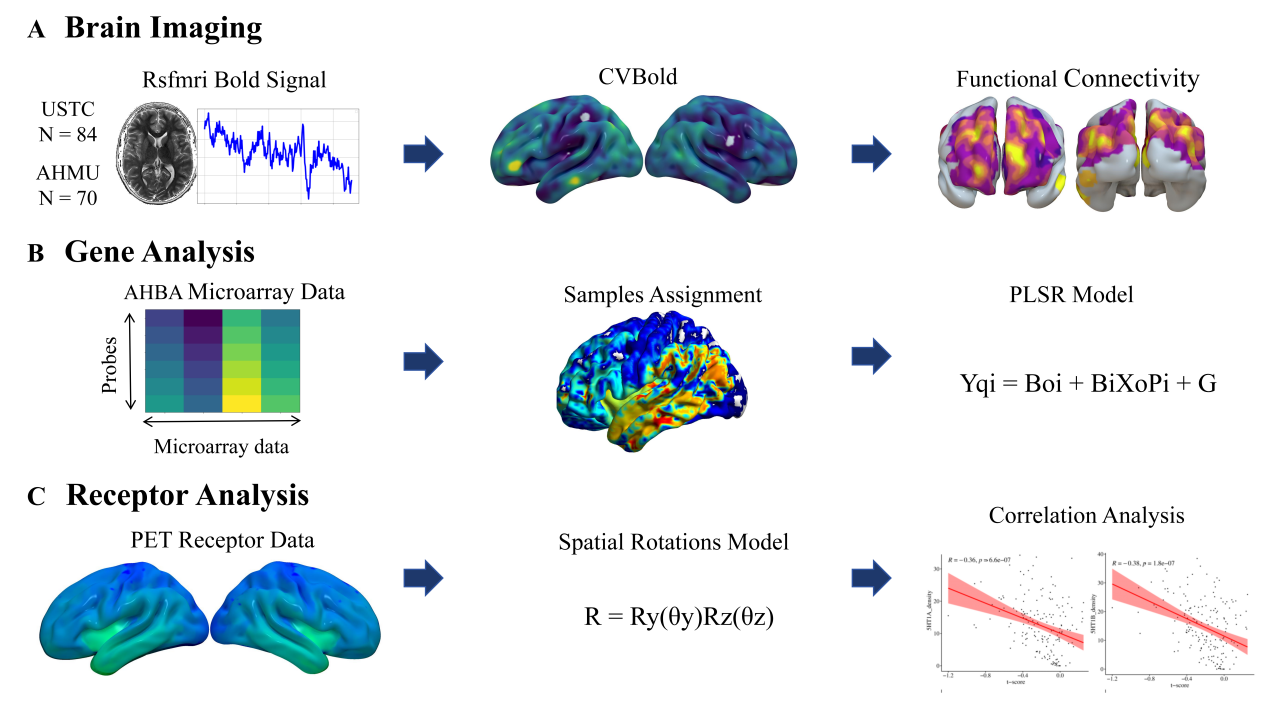


Fig.S4. Data analysis flow chart.
